# Video-Assisted Thoracoscopy is Feasible Under Local Anesthesia

**DOI:** 10.1155/DTE.4.177

**Published:** 1998

**Authors:** Hans J. M. Smit, Franz M. N. H. Schramel, Tom G. Sutedja, Luud S. C. ter Laak-Uytenhaak, Marjolein H. Nannes-Pols, Pieter E. Postmus

**Affiliations:** Department of Pulmonary Medicine Free University Hospital Postbus 7057 Amsterdam MB 1007 The Netherlands

## Abstract

Video-assisted thoracoscopy (VAT) is usually performed under general anesthesia (GA). We performed an analysis to determine whether multithoracoport VAT under local anesthesia (LA) is feasible.

*Methods*: Forty-five VAT under LA were performed in 34 men and 11 women (mean age 46.8 years) in the endoscopy room.

*Results*: The waiting time for VAT under LA was 0.5–6h on working days. There were no major complications during or after the VAT. In 9 patients, pleural malignancy was diagnosed, and in 7 patients suspected malignancy was excluded. In 5 patients we found bacterial empyema, of whom 4 had diathermic adhesiolysis during VAT. In 4 patients, the clinical diagnosis was tuberculosis by exclusion, and in 2 patients no conclusive diagnosis could be drawn. VAT influenced treatment policy 15 times, and in 17 pneumothorax patients talc poudrage was performed during the procedure.

*Conclusion*: VAT under LA is safe, effective, logistically simple, and requires no long waiting times. No conversion to GA was necessary.


*Diagnostic and Therapeutic Endoscopy*, Vol. 4, pp. 177–182
Reprints available directly from the publisher
Photocopying permitted by license only

© 1998 OPA (Overseas Publishers Association)
Amsterdam B.V. Published under license
under the Harwood Academic Publishers imprint,
part of The Gordon and Breach Publishing Group.
Printed in Singapore.

### Video-Assisted Thoracoscopy is Feasible under Local Anesthesia

HANS J.M. SMIT*, FRANZ M.N.H. SCHRAMEL, TOM G. SUTEDJA,
LUUD S.C. TER LAAK-UYTENHAAK, MARJOLEIN H. NANNES-POLS and PIETER E. POSTMUS

*Department of Pulmonary Medicine, Free University Hospital, Postbus 7057, 1007 MB Amsterdam, The Netherlands*

*(Received 16 July 1997; Revised 14 November 1997; In final form 1 December 1997)*

**Video-assisted thoracoscopy (VAT) is usually performed under general anesthesia (GA). We performed an analysis to determine whether multithoracoport VAT under local anesthesia (LA) is feasible.**

***Methods*: Forty-five VAT under LA were performed in 34 men and 11 women (mean age 46.8 years) in the endoscopy room.**

***Results*: The waiting time for VAT under LA was 0.5–6 h on working days. There were no major complications during or after the VAT. In 9 patients, pleural malignancy was diagnosed, and in 7 patients suspected malignancy was excluded. In 5 patients we found bacterial empyema, of whom 4 had diathermic adhesiolysis during VAT. In 4 patients, the clinical diagnosis was tuberculosis by exclusion, and in 2 patients no conclusive diagnosis could be drawn. VAT influenced treatment policy 15 times, and in 17 pneumothorax patients talc poudrage was performed during the procedure.**

***Conclusion*: VAT under LA is safe, effective, logistically simple, and requires no long waiting times. No conversion to GA was necessary.**

*Keywords:* Local anesthesia, Pleural fluid, Pleurodesis, Pneumothorax, Video-assisted thoracoscopy

### INTRODUCTION

Thoracoscopy has been performed as a safe technique under local anesthesia (LA) in Europe on a larger scale since the 1950s [1–5]. Recently improved techniques led to rediscovery of thoracoscopy for diagnostic and therapeutic purposes [6–10]. We noticed a trend in The Netherlands of more thoracoscopies being performed in the operation theater under general anesthesia (GA) with double lumen intubation [9–14]. The reasons might be the newly improved video-assisted imaging equipment or easier access to the operation theater for various surgical disciplines.

We reported that this trend towards thoracoscopies (VAT) under GA has led to prolonged

* Corresponding author. Tel.: 31-20-4444782. Fax: 31-20-4444328.

waiting times, longer hospitalization time, increased costs and the need for the more complex GA with double lumen intubation [15].

The purpose of our analysis was to confirm the feasibility of thoracoscopy under LA using modern video-assisted techniques and multiple thoracoports as a safe and effective technique in a standard endoscopy room. We, therefore evaluated the complications and the diagnostic yield of these thoracoscopies in various pleural diseases, plus the possibility of performing talc poudrage in patients with spontaneous pneumothorax (SP).

### PATIENTS AND METHODS

From March 1995 until April 1996, 45 consecutive VAT procedures were performed in the endoscopy room under LA by two pulmonary physicians, and an assisting nurse (Fig. 1). Indications were: 18 cases of spontaneous pneumothoraces, 23 cases of pleural effusion, and 4 cases of pleural thickening.

In all patients VAT was performed for diagnostic purposes. In case of spontaneous pneumothorax, bullae or other abnormalities were registered. In case of pleural effusion or pleural thickening, parietal biopsies were taken. The VAT had therapeutical implications when adhesions were removed with electrocautery or, in case of spontaneous pneumothorax, patients received talc poudrage.

Patient characteristics are shown in Table I. In patients with pleural fluid, pleural tap preceded the VAT, but failed to provide the diagnosis. Intra parenchymal and mediastinal lesions were not considered to be indications for VAT under LA in this study.

Patients had nothing per mouth for 4 h prior to VAT, received 0.5 mg atropine 0.5 h prior to VAT and received intravenous (iv) saline infusion. Two liters per minute of supplementary oxygen was administered by nasal prongs. Oxygen saturation ($SaO_2$) was monitored with the pulse oximeter. In cases of talc poudrage, 1 ml

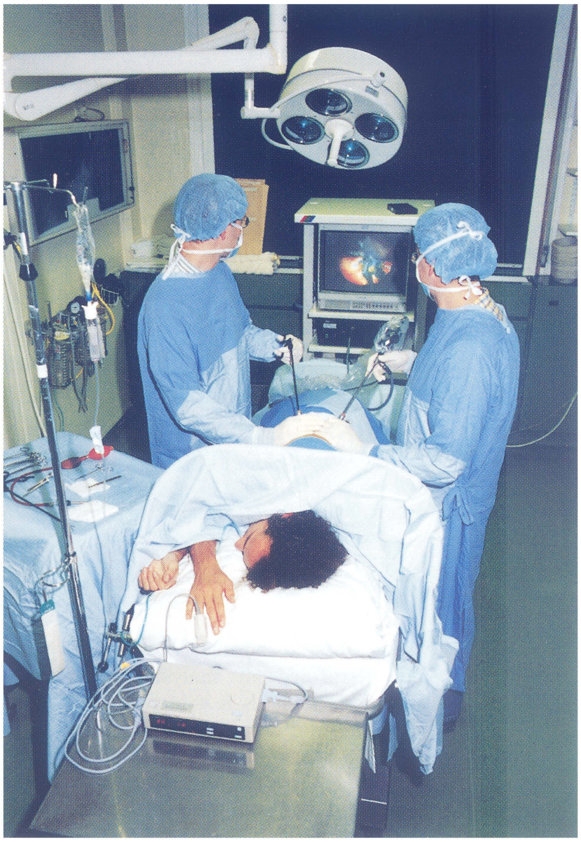

FIGURE 1 VAT procedures were performed in the endoscopy room under local anesthesia by two pulmonary physicians and an assisting nurse.

TABLE I Patients' characteristics ($n = 45$)

| Mean age in years (range) | 46.8 (18–83) |
|---|---|
| Male/female | 34/11 |
| Pleural effusion* | 23 |
| Pneumothorax* | 18 |
| Pleural thickening* | 4 |

*Indication for thoracoscopy.

Thalamonal® (2.5 mg droperidol and 0.05 mg fentanyl) was given iv with 2.5 mg iv bolus of midazolam, followed by a midazolam drip infusion of 0.30 mg per minute. The VAT technique was the same as has been previously published [16,17]. After local anesthesia with < 15 ml of lidocaine 1%, multiple 8 mm trocars were used, when necessary.

Adhesiolysis and hemostasis were performed with electrocautery forceps. Talc poudrage and chest tube insertion were performed under direct vision. Data of all 45 patients were analyzed retrospectively with regard to patient demographics, radiological aspects, VAT findings, post-thoracoscopy care, and results of cytology, histology, and cultures. There was no direct comparison of the diagnostic yield with VAT under GA. For ISP patients hospitalization time, waiting time, and drainage time was compared to recent data of the previous 3 years before, when VAT was performed under GA in all SP patients.

### RESULTS

### VAT Procedure

The waiting time for VAT under LA was 0.5–6 h. VAT was always performed in the endoscopy room. The diathermic forceps was used successfully in 9 patients to perform adhesiolysis, and stop minor bleeding. Seventeen of 18 SP patients received pleurodesis with dry powder insufflation of 3 g talc. The average duration of VAT under LA was 15 min (range 8–30). Patients could return to the ward immediately thereafter.

Pain was never a major problem during the procedure. Thalamonal®, and midazolam in case of talc pleurodesis during VAT, were sufficient. There were no major complications such as major bleeds or respiratory failure during the procedure. No conversion to GA was required.

### Post-VAT Period

After the VAT, patients received paracetamol, ibuprofen, and morphine, on demand. Morphine was no longer necessary after the first 48 h period. Five of the 9 confirmed pleural malignancies received pleurodesis through the chest tube, after the diagnosis was made by VAT. In 5 cases of empyema, chest tubes were used for daily rinsing of the pleural cavity. During the post-VAT period there were no major complications such as pleural or pulmonary infections, atelectasis or re-expansion edema. The mean drainage time was 2.9 days (range 0–9, sd 1.8) (Table II). For patients requiring no further procedures, the mean hospitalization time from VAT on was 4.1 days (range 2–10, sd 1.8).

TABLE II Drainage time after VAT

| Final diagnosis | *n* | Drainage time in days |
|---|---|---|
| Pneumothorax | 18 | 3.0 (2–9) |
| Bacterial empyema | 5 | 16.0 (6–35) |
| Other* | 21 | 2.8 (0–8) |
| Total† | 39 | 2.9 (0–9) |

*Excluding patient with persistent air leakage because of malignant disease.
† Excluding bacterial empyema and excluding *.

The hospitalization time of a consecutive series of SP patients treated with VAT under GA in the previous 3 years was $10.6 \pm$ sd 4.0 on the average, including the waiting time for VAT under GA of $4.4 \pm$ sd 2.5 (mean 6.2 days from VAT on) [15]. Diagnostic findings are shown in Table III. In 6 of the 9 patients with malignant pleurisy, the VAT diagnosis resulted in a change of treatment policy. Two patients received VAT surgery under GA, 2 weeks following VAT under LA, without additional success; one patient with malignant pleural disease had persistent air leakage, and one required additional diagnostic lung biopsies later on.

### DISCUSSION

Thoracoscopy under LA has been performed by the pulmonologist in Europe for decades [1–3,7,8]. This procedure was normally performed by introduction of one relatively thick trocar, through which a rigid scope, together with biopsy scissors, was introduced. The major disadvantage was that a limited area could be inspected, and that lesions covered by the collapsed lung were found with difficulty or not at all. A thick trocar decreases the area within reach, because the angle to the ribs is less favorable. Furthermore, full

TABLE III Final diagnosis, therapy during VAT, diagnostic yield, VAT influence on treatment policy

| Post-VAT diagnosis* | *n* | Pre-VAT diagnosis | Therapeutical VAT | Diagnostic yield | Change in treatment policy |
|---|---|---|---|---|---|
| Pneumothorax | 18 | Pneumothorax | Talc poudrage: 17 | bulla\bleb: 11 | None |
| Malignant pleural Effusion | 9 | Pleural exsudate or pleural thickening | | Pa. compatible with: | Pleurodesis: 5 |
| | | | | Lung cancer: 4<br>Mesothelioma: 3<br>Other malignancy: 2 | Excluded curative treatment: 3<br>Chemotherapy: 3 |
| Reactive pleural effusion | 7 | Malignancy elsewhere with pleural exsudate | | Pleural malignancy excluded | Intended curative treatment executed: 4 |
| Bacterial empyema | 5 | Pleural exsudate | Adhesiolysis: 4 | Positive cultures | Daily pleural lavage and antibiotics: 5 |
| Tuberculosis† | 4 | Pleural exsudate | | Granulomata: 2<br>Positive cultures: 0 | Anti tuberculous drugs: 4 |
| No conclusion | 2 | Pleural exsudate | | None | None |
| Total | 45 | | | None: 4 | None: 6 excluding 18 SP |

The table describes the usefulness of VAT under LA in relation to the final diagnosis, the therapeutical options during the VAT, and the final treatment policy.
*Abbreviations*: Pa. = pathology, SP = idiopathic spontaneous pneumothorax.
*Post-VAT diagnosis includes the other clinical findings.
†Tuberculosis was 2 times based on excluding other disease by VAT and the fact they responded well on anti tuberculous drug therapy.

lung inspection was impossible, because no other manipulating instruments were used.

With the introduction of VAT, it became common practice to introduce multiple trocars enabling manipulation of the lung and the possibility of introducing instruments through a different trocar than the endoscope. The use of thinner trocars was made possible since the instruments were used separately, and were therefore smaller in diameter.

In the United States, and also in Europe, thoracoscopy gained popularity in recent years as VAT under GA performed in the operation theater [9–15,18], which has certain risks [19]. By using more thoracoports with smaller trocars, it became possible to improve the inspection of the pleural cavity and the lung. However, the wish to do the VAT under GA in the operating theater resulted in unacceptable long waiting time, prolonged hospitalization time, and thus, increased costs [15]. Furthermore, in our opinion, the GA and double lumen intubation are unnecessarily uncomfortable for the patient, and more complex than the standard approach.

In a recent Mayo Clinic report of 771 VAT under GA [20], no conversion to thoracotomy was necessary because of complications. The procedure itself seems thus to be quite safe. The conversion to thoracotomy, mentioned in the Mayo Clinic study, was made because of surgical technical reasons in a different group of patients. The patient groups comparable with our indications, in this Mayo Clinic study, had no complications or conversion to GA, as was our own experience. Another advantage of the video-assisted procedure with multiple thin trocars is the improvement in educating residents and students of the anatomy of the pleural cavity and on the technique of thoracoscopy. We, therefore, decided to evaluate the feasibility of this procedure under LA for diagnostic and therapeutic reasons. The indication for visceral and mediastinal biopsies during VAT under LA should only be made by experienced performers, after careful deliberation. We have excluded these indications in our study period.

In our previous series [15], the waiting time for VAT under GA was 4.4 ± sd 2.5 days. In comparison to this data, the present waiting time for VAT under LA is shorter (0.5–6 h on working days). In several reports, the assumption has been made that double lumen intubation provides better assessment of the thoracic cavity, although no direct comparison was made. In our series, we encountered no difficulty creating sufficient space for proper assessment of the pleural cavity. By asking the patient to inhale, the air flows into the pleural cavity through the open trocar. The cooperating patient can help in performing diagnostic tests, such as the Valsalva maneuver to enable bullae visualization, and the sniff test to check the diaphragmatic function both of which are not possible under GA. The mean duration of the VAT procedure under LA was short, and certainly not longer in duration than in VAT under GA [20]. VAT under LA also provides therapeutical interventional options, such as adhesiolysis, coagulation of blebs and bullae, and pleurodesis. We performed talc poudrage in 17 of 18 cases of pneumothorax. The sometimes painful event was well tolerated under the given medication of midazolam and thalamonal®, in part because of the retrograde amnesia.

There were no major complications during or following the procedure. In patients without empyema, the chest tube could be removed after an average of 2.9 days (Table II).

The diagnostic yield in our series was sufficient, although we made no direct comparison with VAT under GA. The influence of VAT on future therapy, as well as the diagnostic yield are shown in Table III.

### CONCLUSION

VAT under LA is safe, effective, logistically simple, and requires no unnecessarily long waiting times. In our series, no conversion to GA was necessary. Because of the reduced waiting time, the hospitalization time for VAT under LA is consistently shorter than under GA. VAT with multiple thoracoports is a feasible procedure under local anesthesia in this category of patients, and should therefore be performed under general anesthesia only when it is not possible to perform it under local anesthesia.

### *References*

[1] Jacobeus, H.C. Ueber die möglichkeiten die Zystoscopie bei Untersuchung seroser Höhlen anzuwenden. *Münchener Medizinischer Zschr* 1910; **40**: 2090–2092.

[2] Swierenga, J., Wagenaar, J.P.M. and Bergstein, P.G.M. The value of thoracoscopy in the diagnosis and treatment of diseases affecting the pleura and lung. *Pneumonologie* 1974; **151**: 11–18.

[3] Vanderschueren, R.G.J.R.A. Le talcage pleural dans le pneumothorax spontanee. *Pouman Coeur* 1981; **37**: 273–276.

[4] Enk, B. and Viskum, K. Diagnostic thoracoscopy. *Eur. J. Respir. Dis.* 1981; **62**: 344–351.

[5] De Camp, P.T., Mosely, P.W., Scott, M.L. and Halch, H.B., Jr. Diagnostic thoracoscopy. *Ann. Thorac. Surg.* 1973; **16**: 79–84.

[6] Janssen, J.P., Postmus, P.E., van Mourik, J.C. and Cuesta, M.A. Diagnostic thoracoscopy. *Diagnostic and Therapeutic Endoscopy* 1995; **1**: 195–200.

[7] Mathur, P.N. and Loddenkemper, R. Medical thoracoscopy: Role in pleural and lung diseases. *Clinics in chest medicine* 1995; **16**(3): 487–496.

[8] Loddenkemper, R. and Boutin, C. Thoracoscopy: present diagnostic and therapeutic indication (review). *Eur. Respir. J.* 1993; **6**: 1544–1555.

[9] Coltharp, W.H., Arnold, J.H., Alford, W.C. *et al.* Videothoracoscopy: improved technique and expanded indications. *Ann. Thorac. Surg.* 1992; **53**: 776–779.

[10] Schramel, F.M.N.H., Sutedja, T.G., Janssen, J.P., Cuesta, M.A., van Mourik, J.C. and Postmus, P.E. Prognostic factors in patients with spontaneous pneumothorax treated with video-assisted thoracoscopy. *Diagnostic and Therapeutic Endoscopy* 1995; **2**: 1–5.

[11] Lewis, R.J., Caccavale, R.J., Sister, G.E. and Mackenzie, J.W. One hundred consecutive patients undergoing video-assisted thoracoscopic operations. *Ann. Thorac. Surg.* 1992; **54**: 421–426.

[12] Wakabayashi, A. Expanded applications of diagnostic and therapeutic thoracoscopy. *J. Thorac. Cardiovasc. Surg.* 1991; **102**: 721–723.

[13] Kaiser, L.R. Video-assisted thoracic surgery; Current state of the art. *Ann. Surg.* 1994; **220**(6): 720–734.

[14] Harris, R.J., Kavuru, M.S., Mehla, A.C. *et al.* The impact of thoracoscopy on the management of pleural disease. *Chest* 1995; **107**(3): 845–852.

[15] Schramel, F.M.N.H., Sutedja, T.G., Braber, J.C.E., van Mouric, J.C. and Postmus, P.E. Cost effectiveness of video-assisted thoracoscopic surgery versus conservative treatment for first time or recurrent spontaneous pneumothorax. *Eur. Respir. J.* 1996; **9**(9): 1821–1825.

[16] Boutin, C., Viallat, J.R. and Aelony, Y. *Practical Thoracoscopy.* Berlin: Springer Verlag 1992.

[17] Mathur, P.N., Astoul, B.S.P. and Boutin, C. Medical thoracoscopy: technical details. *Clin. Chest Med.* 1995; **16**(3): 479–486.

[18] Gharagozloo, F., Read, C. and Wang, K.P. Thoracoscopy in pleural effusion. *J. bronch.* 1996; **3**: 209–216.
[19] Inderbitzi, R.G.C. and Grillet, M.P. Risk and hazards of video-thoracoscopic surgery: a collective review. *Eur. J. Cardio-thorac. Surg.* 1996; **10**: 483–489.
[20] Allen, M.S., Deschamps, C., Jones, D.M., Traslek, V.F. and Pairolero, P.C. Video-assisted thoracic surgical procedures: The Mayo experience. *Mayo Clin. Proc.* 1996; **71**: 351–359.

